# The Majority of Patients Who Undergo ERCP When Large Duct Obstruction Is Evident on Liver Biopsy Have Biliary Findings Amenable to Endoscopic Intervention

**DOI:** 10.3390/jcm12020482

**Published:** 2023-01-06

**Authors:** Melissa Martin, Justin Lee, Roberto Gugig, Andrew Ofosu, Gregory W. Charville, Monique T. Barakat

**Affiliations:** 1 Division of Pediatric Gastroenterology and Hepatology, Stanford University School of Medicine, Stanford, CA 94305, USA; 2 Quantitative Sciences Unit, Department of Medicine, Stanford University, Stanford, CA 94305, USA; 3 Division of Gastroenterology and Hepatology, Stanford University School of Medicine, Stanford, CA 94305, USA; 4 Department of Pathology, Stanford University School of Medicine, Stanford, CA 94305, USA

**Keywords:** biliary obstruction, liver function tests, endoscopic retrograde cholangiopancreatography

## Abstract

(1) Background: Abnormal liver function tests are commonly encountered in clinical practice, often leading to additional workup to determine the underlying etiology of these abnormal laboratory studies. As part of this evaluation, if less invasive imaging studies are performed and are without evidence of biliary obstruction, liver biopsy may be performed, and the finding of large duct obstruction on liver biopsy is commonly encountered. The utility of endoscopic retrograde cholangiopancreatography (ERCP) for evaluation and management of possible biliary obstruction in patients with large duct obstruction on liver biopsy has not been studied to date. (2) Methods: To assess the utility of ERCP in patients with large bile duct obstruction on liver biopsy, we retrospectively evaluated patients with large duct obstruction on liver biopsy from 2010–2019 at our tertiary care and transplant center. Demographic and clinical characteristics were evaluated for all patients, with sub-group analysis for patients who underwent ERCP and those who had intervenable findings at the time of ERCP. Descriptive statistics with proportions, means, and standard deviations were performed for demographics and clinical variables using absolute standardized difference. (3) Results: During the study period, 189 liver biopsies with evidence of large duct obstruction were performed. After exclusion criteria were applied, 166 unique patients were eligible for the study. Ninety-one patients with evidence of large duct obstruction on liver biopsy underwent ERCP and 75 did not. Of the 91 patients who underwent ERCP, 76 patients (84%) had an intervenable finding at ERCP. Patients who underwent ERCP were overall more likely to have had a liver transplant (65% ASD 0.63), have previously undergone cholecystectomy (80%, ASD 0.56), and be immunocompromised (80%, ASD 0.56). (4) Conclusions: ERCP is high yield when large duct obstruction is apparent on liver biopsy, with the majority of patients (84%) who undergo ERCP in this clinical context having a biliary finding necessitating therapeutic endoscopic intervention.

## 1. Introduction

Liver biopsy is part of the typical algorithm for workup of abnormal liver function tests (LFTs) and is typically performed after other less invasive laboratory and imaging studies are unrevealing. Liver histologyhas been found to be of high utility in both the diagnosis and management of these patients with abnormal LFTs and can help determine whether bile formation or flow of bile underlies the problem [1,2].

When large duct obstruction is identified on liver biopsy, additional focused imaging is often performed to evaluate for an obstructing lesion, stone, or stricture [3]. There is lack of consensus regarding which imaging modality is of highest utility balanced with cost effectiveness in this clinical scenario [3], with imaging ranging from abdominal ultrasound (US) and computed tomography (CT) scan to magnetic resonance cholangiopancreatography (MRCP). If definitive biliary obstruction is evident on imaging, then the decision to proceed to endoscopic retrograde cholangiopancreatography (ERCP) for therapeutic biliary intervention is relatively straightforward. However, in the absence of definitive imaging-based evidence of biliary obstruction, the utility of ERCP is less certain and has not been rigorously studied.

Many ERCPs are undertaken for further evaluation of the finding of large duct obstruction on liver biopsy, and this practice affects thousands of patients nationwide, yet it lacks an evidence basis. ERCP is a relatively high-risk procedure, with a subset of patients experiencing post-procedure adverse events, including pancreatitis, bleeding, infection, and perforation [4]. While these risks are acceptable if ERCP positively impacts diagnosis and therapy, data surrounding the risk:benefit relationship of ERCP performed for evaluation of large duct obstruction on liver biopsy is currently lacking. Validation of this indication for ERCP or definitive demonstration that ERCP is not beneficial in this clinical scenario is essential. In the present study, for the first time, we assess the utility of ERCP for patients when histologic features of large bile duct obstruction are present on liver biopsies, and we explore clinical characteristics that might help predict which of these patients would benefit most from ERCP.

## 2. Methods

### 2.1. Study Design and Data Source

We performed a retrospective evaluation of adult patients with large duct obstruction on liver biopsy from 2010–2019 at Stanford Hospital, a major referral and transplant center in the Western United States. The study was approved by the Stanford IRB (Protocol # 57500). Patients whose liver biopsy was performed at the time of native liver explant were excluded from the analysis. For patients who had more than one liver biopsy, only the first liver biopsy was included in the analysis. Histologic diagnoses were rendered by subspecialized hepatobiliary pathologists using hematoxylin and eosin, trichrome, rhodanine, and diastase-predigested periodic acid-Schiff stains to assess for standard morphologic features of large duct obstruction, including portal edema, ductular proliferation, neutrophilic pericholangitis, canalicular/ductal cholestasis, reactive epithelial changes of the interlobular bile ducts, and acute cholangitis. Features of prolonged obstruction, such as fibrosis, feathery degeneration, and periportal copper accumulation, were also examined. Each patient record was thoroughly reviewed by two reviewers, and the following covariates were collected for each patient: age, gender (female/male), race (white, Asian, other), ethnicity (Hispanic/non-Hispanic), insurance status (private insurance, government insurance, unknown), body mass index (BMI), history of cholecystectomy, hepatic comorbidities, malignancy, metastatic lesions, abdominal trauma, immunocompromised, liver transplant, interval time from liver transplant to biopsy if applicable (1 month or less, 1 year or less, 2 years or less, 3 years or more), imaging performed (abdominal US, CT scan, MRCP), history of ERCP prior to liver biopsy, ERCP after liver biopsy, and labs (total bilirubin, Gamma-glutamyl transferase (GGT), alkaline phosphatase (ALP), aspartate aminotransferase (AST), alanine aminotransferase (ALT), platelets, and international normalized ratio (INR)). Only patients who had no definitive obstructive findings on imaging studies prior to liver biopsy and ERCP were included in this study. For each of the imaging modalities, we specified whether the documented CBD diameter was <4 mm, 4–7 mm, 8–10 mm, >10 mm. ERCPs were categorized as positive or negative, depending on whether there was an intervenable finding at the time of ERCP. ERCPs with intervenable findings were categorized as ‘positive,’ and findings classified as ‘intervenable’ included: biliary stricture or intrinsic/extrinsic obstructing lesion, biliary stone, papillary stenosis or ampullary obstruction, and anatomical irregularity of the bile duct for which stent placement was necessary.

### 2.2. Study Outcomes

The primary study outcome was the presence of intervenable ERCP findings in patients who had evidence of large duct obstruction on liver biopsy, who subsequently underwent ERCP. The covariates listed above were included in the analysis to determine whether there were clinical differences in patients who had intervenable ERCP findings vs. those who did not.

### 2.3. Statistical Analysis

Descriptive statistics with proportions, means, and standard deviations were presented for the entire cohort in regard to demographics and clinical variables, including prior medical history, labs, and imaging. The study population was stratified by those who underwent ERCP post liver biopsy and those who did not. We evaluated each variable using the absolute standardized difference (ASD). ASD is defined as the difference in means or proportions divided by the pooled standard deviation. ASD of 0.2, 0.5, and 0.8 represent small, median, and large differences. ASD values < 0.2 represent no discernable difference between the two groups. The larger the ASD, the more different the two groups are from each other. ASD is increasingly utilized for analysis of descriptive data and quantification of differences between groups, while *p*-values are largely reserved for hypothesis testing [5]. *p*-values indicate whether a difference exists, but do not describe the difference of the effect size [6]. ASD is a measure of covariate balance [7].

The study population that underwent ERCP was then stratified by those who had a positive ERCP and those who had a negative ERCP. Positive ERCP was defined as an ERCP in which there was an intervenable finding. The ASD was also calculated for this group. Lastly, we performed an exploratory univariate analysis to determine whether specific patient characteristics, imaging results, and laboratory findings were sufficient to predict whether an ERCP would result in the presence of an intervenable lesion (positive ERCP outcome).

## 3. Results

### 3.1. Demographic and Clinical Characteristics of All Patients

**Figure 1:**
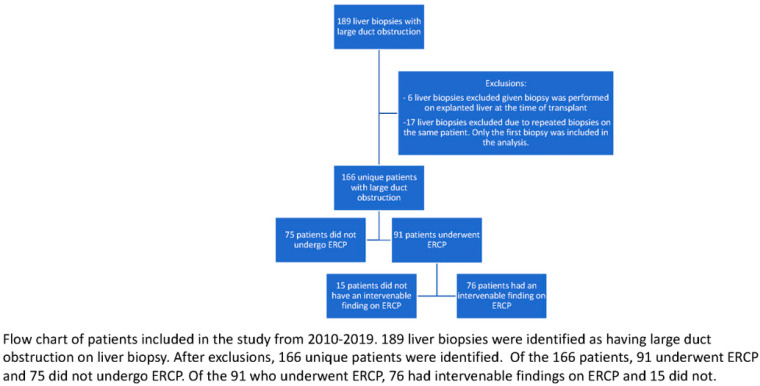
Study flow diagram.

During the study period from 2010–2019, 189 liver biopsies with evidence of large duct obstruction were identified (Figure 1). Six patients were excluded from the analysis, given that their liver biopsy was performed on their explanted liver at the time of transplant. For patients with multiple liver biopsies showing large duct obstruction, only the first liver biopsy was included in the analysis. After exclusion criteria were applied, 166 unique patients were identified as having large duct obstruction on liver biopsy (Figure 1).

Table 1 depicts demographic and clinical characteristics of the study population. The mean patient age was 54 years (SD 14.29). Males accounted for the majority of patients (63%). Racial composition was notable for 59% white and 17% Asian patients. The study population was predominantly non-Hispanic (72%). The mean GGT and total bilirubin were 418 U/L and 4.5 mg/dL, respectively. Approximately 50% of patients were post-liver transplantation at the time of the liver biopsy and ERCP and, of those patients, nearly 50% underwent liver biopsy within the first month following transplantation. The majority of patients had undergone previous cholecystectomy (70%) and were immunocompromised (72%). Immunocompromised status was primarily due to history of liver transplantation or malignancy. The most common imaging modality used to evaluate for biliary obstruction was abdominal US (83%), followed by CT scan (55%) and MRCP (50%). For all three imaging modalities, in the majority of cases, the CBD diameter was less than 7 mm. Prior to undergoing ERCP, the CBD diameter was 8 mm or larger in only 18% of patients who underwent abdominal US, 16% of those who underwent CT scan, and 14% of those who underwent MRCPs.

### 3.2. Demographic and Clinical Characteristics of Patients Who Underwent ERCP

#### 3.2.1. Demographics

During the study period, 91 patients with evidence of large duct obstruction on liver biopsy underwent ERCP and 75 did not (Table 2). Among patients who underwent ERCP, 65% were white and 12% were Asian, as compared to 52% white and 23% Asian in the non-ERCP group (ASD = 0.32).

#### 3.2.2. Clinical Characteristics

The GGT and ALP were noted to be significantly higher in the ERCP group, with ASD 0.33 and 0.48, respectively. The mean GGT for the ERCP group was 481 U/L (SD 601.88), compared to 319 U/L (SD 335.37) for the non-ERCP group. ALP showed a similar trend, with mean of 441 U/L (SD 416.32) for the ERCP group and mean of 282 U/L (SD 219.41) for the non-ERCP group. In the non-ERCP group, 59% of patients who underwent liver transplantation had undergone liver biopsy within the first month following the liver transplant, as compared to 43% in the ERCP group (ASD 0.50). Patients who underwent ERCP were overall more likely to have undergone prior liver transplant (65%), have previously undergone cholecystectomy (80%), and be immunocompromised (80%) (Table 2). Compared to patients who did not undergo ERCP, patients who underwent ERCP were more likely to have imaging findings notable for a CBD diameter >10 mm, regardless of imaging modality [abdominal US: 11% vs. 6% (ASD 0.35), CT scan: 18% vs. 2% (ASD 0.56), MRCP: 10% vs. 3% (ASD 0.36)].

### 3.3. Demographic and Clinical Characteristics of Patients with an Intervenable Lesion at ERCP

#### 3.3.1. Demographics

Of the 91 patients who underwent ERCP, 76 patients (84%) had an intervenable finding at ERCP. Sixty-eight percent of these patients with an intervenable finding at ERCP were male, while only 32% were female (ASD 0.60). Among patients who underwent ERCP without an intervenable finding, 67% were white and 27% were Asian, compared to 65% white and 9% Asian in the positive ERCP group (ASD 0.67). The majority of patients in the positive ERCP group were non-Hispanic (66%). Insurance status had a large ASD (0.77) between the ERCP positive and negative groups, with private, government, and unknown insurance accounting for 2%, 24%, and 62%, respectively, in the ERCP positive group and 47%, 20%, and 33%, respectively, in the non ERCP group. The positive ERCP group had higher rates of unknown insurance status.

#### 3.3.2. Clinical Characteristics

Laboratory studies of the 76 patients with large duct obstruction on liver biopsy who had an intervenable finding at ERCP (positive ERCP group) were notable for significantly higher ALP in comparison with patients who did not have an intervenable finding at ERCP (ASD 0.47, Table 3). CBD diameter greater than 8 mm was also more commonly found in the ERCP-positive group relative to the ERCP-negative group (abdominal US: 26% vs. 8% (ASD 1.23), MRCP: 16% vs. 0% (ASD 0.81), CT scan: 26% vs. 0% (ASD 1.11)). Patients in the positive ERCP group were more likely to have had a cholecystectomy, have previously undergone liver transplantation, and be immunocompromised. These characteristics (cholecystectomy, immunosuppression, prior liver transplant) are associated with liver transplant recipients, and the findings are driven by this liver transplant population.

Among the 76 patients who had intervenable findings during ERCP, the three most common interventions were stent placement for benign biliary stricture (41), sphincterotomy with stone extraction for choledocholithiasis (16), and stent placement for malignant biliary stricture (12).

## 4. Predictors of Intervenable Lesion Detectable at ERCP

Lastly, we performed an exploratory univariate analysis to determine a model for positive ERCP as the outcome. As shown in Table 4, males are seemingly more likely to have a positive ERCP finding than females, with an odds ratio (OR) of 3.25 with *p*-value of 0.04. Additionally, patients in this cohort with government insurance and unknown insurance status had ORs of 3.82 and 5.98, and they were much more likely to have positive ERCP findings when compared to patients with private insurance, with *p*-values < 0.05. History of cholecystectomy, liver transplant, and immunocompromised status all had statistically significant ORs, with *p*-values less than 0.05, indicating these factors were predictive of finding intervenable lesions at ERCP in the setting of large duct obstruction on liver biopsy.

## 5. Adverse Events Associated with ERCP

Among the 91 patients who underwent ERCP, 2 (2.2%) developed mild post-ERCP pancreatitis, characterized by abdominal pain and elevated lipase. Post-ERCP pancreatitis symptoms had resolved within 2 days of the procedure for both patients, and patients were without any long-term sequelae (e.g., pancreatic fluid collections/necrosis, infection) associated with the post-ERCP pancreatitis episodes. None of the patients who underwent ERCP developed the post-ERCP adverse events of infection, bleeding, or perforation.

## 6. Discussion

Our study found that, among patients who had no definitive imaging evidence of biliary obstruction and who then underwent ERCP in the setting of large duct obstruction on liver biopsy, the vast majority (84%) had intervenable findings at ERCP. The biliary interventions performed to address obstruction included sphincterotomy, balloon dilatation, stone removal, or stent placement at the time of the procedure. This is the first study to evaluate the endoscopic implications of large duct obstruction on liver biopsy and to highlight the utility of ERCP when large duct obstruction is apparent on liver biopsies. Importantly, this study focuses on the challenging clinical scenario of patients who had no evidence of biliary obstruction on cross-sectional imaging, and ERCP was performed based primarily on liver biopsy demonstrating large duct obstruction. ERCP is among the most complex endoscopic procedures and is associated with significant risks, including post-ERCP pancreatitis, bleeding, and infection. The present study validates histologic evidence of large duct obstruction as an indication for ERCP and suggests that potentially more patients with large duct obstruction on liver biopsy might benefit from ERCP, even in the absence of obstructive findings on imaging.

The decision process surrounding if and when to perform an ERCP in a given patient often involves an interplay between multiple providers, comprising inpatient and outpatient care teams [8]. The specific mix of clinical teams involved in a patient’s care depends on a given patient’s medical co-morbidities and the tempo, characteristics, and severity of symptoms that led to the patient’s clinical presentation [9]. This unpredictability in the decision-making process leading up to outpatient referral or inpatient consultation for ERCP is unfortunate, and it leads to substantial heterogeneity in practice patterns, as well as potentially compromised care. The present outcomes study represents a type of study that has the potential for substantial impact on patient care and outcomes. Indications for ERCP should be assessed rigorously to either validate those indications or provide evidence that specific disorders or findings do not represent valid indications for ERCP. For example, for some ERCP indications, the absence of viable medical therapies, the plausibility that ERCP may improve the disease course, and the impression among care team members that other institutions or endoscopists typically perform ERCP for a given indication sometimes couple with patient and care team ‘desperation,’ resulting in decades of performance of ERCP for indications that are not evidence based. A key example of this phenomenon is Sphincter of Oddi dysfunction, for which performance of ERCP was routine worldwide, without evidence-based support for its practice. The EPISOD study evaluated the practice and potential risk/benefit of ERCP with sphincterotomy for patients with sphincter of Oddi dysfunction and irrefutably demonstrated that ERCP should not be performed for patients with sphincter of Oddi dysfunction type III (abdominal pain alone) [10].

The present study is the first of its kind to evaluate the utility of ERCP in patients with large duct obstruction on liver biopsy, without evidence of biliary obstruction on cross-sectional imaging. We first characterized all patients who had undergone ERCP following a liver biopsy diagnosis of large duct obstruction and then compared sub-groups based on ERCP outcome and benefit. The vast majority of these patients who had large duct obstruction on liver biopsy and underwent ERCP had intervenable findings at ERCP, which would validate performance of ERCP in this population. Patient characteristics associated with these high-utility ERCPs with interventions to address the cause of biliary obstruction included substantial elevation in ALP and biliary dilation greater than 8 mm on imaging. Patients with intervenable lesions at ERCP were also more likely to have a history of liver transplantation and cholecystectomy, and to be immunosuppressed. These patient characteristics may be used, along with the clinical context of presentation and clinical trajectory, to guide the timing and performance of ERCP for further evaluation of histologic features of large duct obstruction on biopsy. Our demographic characterization revealed that white patients were more likely to undergo ERCP relative to patients of other ethnicities, and patients with intervenable findings on ERCP were primarily male, white, and non-Hispanic. Insurance status also differed significantly between the ERCP positive and negative groups, with the positive ERCP group notable for fewer patients with private insurance (15%). These insurance status results, however, are somewhat limited by the substantial proportion of patients for whom insurance status was unknown in the overall patient sample.

Our study has some noteworthy limitations. First, the sample size for the ERCP negative group was small. We performed an exploratory univariate analysis to determine whether specific patient characteristics, imaging results, and laboratory findings were sufficient to predict whether an ERCP would result in a positive outcome. These findings would then allow us to develop a multivariable logistic regression model to predict a positive ERCP outcome. Given the small sample size of the negative ERCP group, the multivariable model was unreliable due to large confidence intervals and unrealistic z statistics. The results show a univariate association between positive ERCP outcome and cholecystectomy, history of liver transplantation, and immunocompromised status. However, given the small sample size, definitive conclusions cannot be drawn from these exploratory results, but this may inform future studies designed to assess these three covariates in more depth. A second limitation is that all patients included in this study were from one tertiary care center. Thus, it may be the case that different centers across the nation may have different results. Additionally, our patient population was primarily white (59%) and non-Hispanic (72%); hence, it did not reflect the overall diversity of the U.S. population. Finally, the authors wish to underscore that this study is retrospective and subject to limitations of retrospective chart review. In this type of study, it is not possible to assess the relative risk/benefits profile of ERCP for patients with large duct obstruction diagnosed on liver biopsy. Rather, this is a manuscript that retrospectively evaluates findings for patients who underwent ERCP when large duct obstruction was evident on liver biopsy. This study is not intended to assess risks/benefits of ERCP in this clinical scenario, but rather to report findings of ERCP when it is performed in the clinical scenario of large duct obstruction in a tertiary care academic center.

In conclusion, the present study is the first of its kind to evaluate the utility of ERCP after large duct obstruction is diagnosed on liver biopsy. We demonstrate that, even when cross-sectional imaging that is highly sensitive for detecting biliary obstruction shows no definitive evidence of biliary obstruction, most patients with large duct obstruction who undergo ERCP benefit from therapeutic endoscopic intervention. These results represent an initial step toward validating large duct obstruction on liver biopsy as an independent ERCP indication. Future studies including a larger sample size and patients from multiple institutions of variable acuity and tertiary/primary care status would be highly informative. A multi-center study would also enable evaluation of patient sub-groups with large duct obstruction on liver biopsy, such as those who have a history of liver transplant with cholecystectomy and are immunosuppressed, to assess whether other clinical parameters specific to these patient populations may be predictive of higher or lower utility of ERCP. Our study serves as the initial foundation for potential prospective studies focused on ERCP performed for patients with large duct obstruction on biopsy. More broadly, this study may serve as a model for initial evaluation and validation of other indications, for which ERCP is commonly performed although little to no data exist regarding the risk/benefit profile of the specific indication for ERCP.

## Figures and Tables

**Table 1:** Demographics and clinical characteristics of all adult patients with large duct obstruction on liver biopsy.

		Overall
		N = 166
Age (mean (SD))		54.63 (14.29)
Gender n (%)	Female	62 (37)
	Male	104 (63)
Race n (%)	White	97 (59)
	Asian	28 (17)
	Other	39 (24)
Ethnicity n (%)	Non-Hispanic	119 (72)
	Hispanic	46 (28)
Insurance n (%)	Private	23 (14)
	Government	25 (15)
	Unknown	118 (71)
BMI kg/m^2^ (mean (SD))		25.17 (5.00)
***Labs***		
Total bilirubin mg/dL (mean (SD))		4.55 (6.16)
GGT U/L (mean (SD))		418.98 (519.34)
ALP U/L (mean (SD))		369.67 (349.99)
AST U/L (mean (SD))		117.71 (182.23)
ALT U/L (mean (SD))		143.95 (204.75)
Platelet × 10^9^/L (mean (SD))		185.76 (100.08)
INR (mean (SD))		1.20 (0.26)
***Medical History***		
History of liver transplant n (%)	No	79 (48)
	Yes	87 (52)
Interval time from liver transplant to biopsy n (%)	1 month or less	43 (48)
	1 year or less	31 (34)
	2 years or less	3 (3)
	3 years or more	13 (14)
Immunocompromised status n (%)	No	46 (28)
	Yes	120 (72)
History of cholecystecomy n (%)	No	49 (30)
	Yes	117 (71)
History of abdominal trauma n (%)	No	162 (98)
	Yes	3 (2)
History of hepatic or biliary metastatatic lesions n (%)	No	157 (95)
	Yes	8 (5)
History of other malignancy n (%)	No	153 (92)
	Yes	13 (8)
History of comorbidiites n (%)	None	26 (16)
	Stone disease	5 (3)
	Cirrhosis	11 (7)
	Malignancy	16 (10)
	Other	108 (65)
History of prior ERCP n (%)	No	124 (75)
	Yes	42 (25)
***Imaging***		
Abdominal US, CBD diameter n (%)	Not performed	28 (21)
	<4 mm	41 (30)
	4–7 mm	41 (31)
	8–10 mm	12 (9)
	>10 mm	11 (8)
MRCP, CBD diameter n (%)	Not performed	83 (65)
	<4 mm	26 (20)
	4–7 mm	7 (6)
	8–10 mm	3 (2)
	>10 mm	9 (7)
CT scan, CBD diameter n (%)	Not performed	74 (70)
	<4 mm	17 (16)
	4–7 mm	0 (0)
	8–10 mm	4 (4)
	>10 mm	11 (10)

**Table 2:** Demographics and clinical characteristics stratified by whether ERCP was performed.

		No ERCP	ERCP	ASD
		N = 75	N = 91	
Age (mean (SD))		53.93 (15.09)	55.22 (13.65)	0.09
Gender n (%)	Female	29 (39)	33 (36)	0.05
	Male	46 (61)	58 (64)	
Race n (%)	White	38 (52)	59 (65)	0.32
	Asian	17 (23)	11 (12)	
	Other	18 (25)	21 (23)	
Ethnicity n (%)	Non-Hispanic	55 (74)	64 (70)	0.09
	Hispanic	19 (26)	27 (30)	
Insurance n (%)	Private	5 (7)	18 (20)	0.74
	Government	4 (5)	21 (23)	
	Unknown	66 (88)	52 (57)	
BMI kg/m^2^ (mean (SD))		25.09 (5.20)	25.23 (4.86)	0.03
***Labs***				
Total bilirubin mg/dL (mean (SD))		4.11 (6.19)	4.90 (6.16)	0.13
GGT U/L (mean (SD))		319.00 (335.37)	481.93 (601.88)	0.33
ALP U/L (mean (SD))		282.41 (219.67)	441.59 (416.32)	0.48
AST U/L (mean (SD))		117.95 (200.58)	117.51 (166.73)	0
ALT U/L (mean (SD))		153.30 (208.02)	136.44 (202.92)	0.08
Platelet × 10^9^/L (mean (SD))		199.23 (111.34)	174.81 (89.02)	0.24
INR (mean (SD))		1.23 (0.32)	1.17 (0.19)	0.24
***Medical History***				
History of liver transplant n (%)	No	47 (63)	32 (35)	0.57
	Yes	28 (37)	59 (65)	
Interval time from liver transplant to biopsy n (%)	1 month or less	17 (59)	26 (43)	0.5
	1 year or less	7 (24)	24 (39)	
	2 years or less	0 (0)	3 (5)	
	3 years or more	5 (17)	8 (13)	
Immunocompromised status n (%)	No	28 (37)	18 (20)	0.4
	Yes	47 (63)	73 (80)	
History of cholecystecomy n (%)	No	31 (41)	18 (20)	0.48
	Yes	44 (59)	73 (80)	
History of abdominal trauma n (%)	No	73 (99)	89 (98)	0.06
	Yes	1 (1)	2 (2)	
History of hepatic or biliary metastatatic lesions n (%)	No	73 (97)	84 (93)	0.19
	Yes	2 (3)	6 (7)	
History of other malignancy n (%)	No	65 (87)	88 (97)	0.37
	Yes	10 (13)	3 (3)	
History of comorbidiites n (%)	None	17 (23)	9 (10)	0.55
	Stone disease	4 (5)	1 (1)	
	Cirrhosis	7 (9)	4 (4)	
	Malignancy	8 (11)	8 (9)	
	Other	39 (52)	69 (76)	
History of prior ERCP n (%)	No	65 (87)	59 (65)	0.53
	Yes	10 (13)	32 (35)	
***Imaging***				
Abdominal US, CBD diameter n (%)	Not performed	17 (25)	11 (17)	0.35
	<4 mm	20 (30)	21 (32)	
	4–7 mm	23 (34)	18 (28)	
	8–10 mm	4 (6)	8 (12)	
	>10 mm	4 (6)	7 (11)	
MRCP, CBD diameter n (%)	Not performed	38 (63)	45 (66)	0.36
	<4 mm	15 (25)	11 (16)	
	4–7 mm	4 (7)	3 (4)	
	8–10 mm	1 (2)	2 (3)	
	>10 mm	2 (3)	7 (10)	
CT scan, CBD diameter n (%)	Not performed	39 (78)	35 (63)	0.56
	<4 mm	8 (16)	9 (16)	
	4–7 mm	0 (0)	0 (0)	
	8–10 mm	2 (4)	2 (4)	
	>10 mm	1 (2)	10 (18)	

**Table 3:** Demographics and clinical characteristics of patients who underwent ERCP stratified by ERCP positive vs ERCP negative.

		Negative ERCP	Positive ERCP	ASD
		N = 15	N = 76	
Age (mean (SD))		52.33 (17.13)	55.82 (12.87)	0.23
Gender n (%)	Female	9 (60)	24 (32)	0.6
	Male	6 (40)	52 (68)	
Race n (%)	White	10 (67)	49 (65)	0.67
	Asian	4 (27)	7 (9)	
	Other	1 (7)	20 (26)	
Ethnicity n (%)	Non-Hispanic	14 (93)	50 (66)	0.73
	Hispanic	1 (7)	26 (34)	
Insurance n (%)	Private	7 (47)	11 (15)	0.77
	Government	3 (20)	18 (24)	
	Unknown	5 (33)	47 (62)	
BMI kg/m^2^ (mean (SD))		25.47 (6.27)	25.17 (4.49)	0.05
***Labs***				
Total bilirubin mg/dL (mean (SD))		4.39 (8.57)	5.00 (5.63)	0.09
GGT U/L (mean (SD))		504.75 (312.00)	480.10 (621.15)	0.05
ALP U/L (mean (SD))		302.20 (257.55)	469.11 (436.98)	0.47
AST U/L (mean (SD))		96.00 (102.59)	121.75 (176.87)	0.18
ALT U/L (mean (SD))		102.13 (65.49)	143.21 (219.84)	0.25
Platelet × 10^9^/L (mean (SD))		203.93 (102.57)	169.07 (85.69)	0.37
INR (mean (SD))		1.15 (0.23)	1.17 (0.18)	0.13
***Medical History***				
History of liver transplant n (%)	No	9 (60)	23 (30)	0.63
	Yes	6 (40)	53 (70)	
Interval time from liver transplant to biopsy n (%)	1 month or less	1 (17)	25 (46)	0.74
	1 year or less	3 (50)	21 (38)	
	2 years or less	1 (17)	2 (4)	
	3 years or more	1 (17)	7 (13)	
Immunocompromised status n (%)	No	6 (40)	12 (16)	0.56
	Yes	9 (60)	64 (84)	
History of cholecystecomy n (%)	No	6 (40)	12 (16)	0.56
	Yes	9 (60)	64 (84)	
History of abdominal trauma n (%)	No	15 (100)	74 (97)	0.23
	Yes	0 (0)	2 (3)	
History of hepatic or biliary metastatatic lesions n (%)	No	14 ( 93)	70 (93)	<0.00
	Yes	1 (7)	5 (7)	
History of other malignancy n (%)	No	14 (93)	74 (97)	0.19
	Yes	1 (7)	2 (3)	
History of comorbidiites n (%)	None	3 (20)	6 (8)	0.74
	Stone disease	1 (7)	0 (0)	
	Cirrhosis	1 (7)	3 (4)	
	Malignancy	0 (0)	8 (11)	
	Other	10 (67)	59 (78)	
History of prior ERCP n (%)	No	12 (80)	47 (62)	0.41
	Yes	3 (20)	29 (38)	
***Imaging***				
Abdominal US, CBD diameter n (%)	Not performed	0 (0)	11 (21)	1.23
	<4 mm	8 (67)	13 (25)	
	4–7 mm	3 (25)	15 (28)	
	8–10 mm	1 (8)	7 (13)	
	>10 mm	0 (0)	7 (13)	
MRCP, CBD diameter n (%)	Not performed	7 (58)	38 (68)	0.81
	<4 mm	4 (33)	7 (13)	
	4–7 mm	1 (8)	2 (4)	
	8–10 mm	0 (0)	2 (4)	
	>10 mm	0 (0)	7 (13)	
CT scan, CBD diameter n (%)	Not performed	5 (56)	30 (64)	1.11
	<4 mm	4 (45)	5 (11)	
	4–7 mm	0 (0)	0 (0)	
	8–10 mm	0 (0)	2 (4)	
	>10 mm	0 (0)	10 (21)	

**Table 4:** Univariate analysis for positive ERCP outcome.

Outcome: ERCP Results Positive (Yes/No)		
Covariates:	OR (95% CI)	*p*-value
Age	1.02 (0.98, 1.06)	0.37
Gender (ref = Female)	3.25 (1.05, 10.7)	0.04
Race grouped (ref = White)		
Asian	0.36 (0.09, 1.57)	0.15
Other (includes Black, American Indian or Alaska Native, Native Hawaiian or Pacific Islander, Other)	4.08 (0.71, 77.39)	0.19
Ethnicity (ref = Non-Hispanic)	7.28 (1.35, 135.54)	0.06
Insurance status (ref = Private)		
Government	3.82 (0.87, 20.75)	0.09
Unknown	5.98 (1.62, 23.86)	0.01
BMI	0.99 (0.88, 1.11)	0.83
Total bilirubin	1.02 (0.93, 1.14)	0.72
GGT	1 (1, 1)	0.94
ALP	1 (1, 1)	0.15
AST	1 (1, 1.01)	0.59
ALT	1 (1, 1.01)	0.48
Platelets	1 (0.99, 1)	0.17
INR	2.34 (0.12, 85.26)	0.61
Liver transplant (ref = No)	3.46 (1.12, 11.4)	0.03
Interval from liver transplant to biopsy (ref = 1 month or less)		
1 year or less	0.28 (0.01, 2.37)	0.29
2 years or less	0.08 (0, 2.52)	0.11
3 years or more	0.28 (0.01, 7.68)	0.39
prior to tranplant	NA	NA
Immunocompromised (ref = No)	3.56 (1.03, 11.86)	0.04
Cholecystectomy (ref = No)	3.56 (1.03, 11.86)	0.04
Abdominal trauma (ref = No)	3172573.13 (0, NA)	0.99
Hepatic or biliary metastatatic lesions (ref = No)	1 (0.15, 19.93)	1
Other malignancy (ref = No)	0.38 (0.03, 8.47)	0.44
Comorbidity (ref = None)		
Stone disease	0 (NA, Inf)	1
Cirrhosis	1.5 (0.12, 37.87)	0.77
Malignancy	21272406.17 (0, NA)	0.99
Other	2.95 (0.55, 13.28)	0.17
Prior ERCP (ref = No)	2.47 (0.71, 11.5)	0.19
Abdominal US, CBD diameter (ref = Not performed)		
<4 mm	0 (NA, 1.29729561707316e+70)	1
4–7 mm	0 (NA, 2.41845124689416e+95)	1
8–10 mm	0 (NA, 3.38586016497201e+95)	1
>10 mm	1 (0, 2.8119434863408e+77)	1
MRCP, CBD diameter (ref = Not performed)		
<4 mm	0.32 (0.07, 1.49)	0.13
4–7 mm	0.37 (0.03, 8.55)	0.44
8–10 mm	21303724.99 (0, NA)	1
>10 mm	21303724.98 (0, NA)	1
CT scan, CBD diameter(ref = Not performed)		
<4 mm	0.21 (0.04, 1.08)	0.06
8–10 mm	19274798.86 (0, NA)	1
>10 mm	19274798.88 (0, NA)	0.99

## Data Availability

Data are unavailable for sharing due to privacy or ethical restrictions.
